# Low reversed cyclic loading tests for integrated precast structure of lightweight wall with single-row reinforcement under a lightweight steel frame

**DOI:** 10.1098/rsos.180321

**Published:** 2018-10-03

**Authors:** Jia Suizi, Cao Wanlin, Zhang Yuchen

**Affiliations:** 1 China University of Geosciences (Beijing), School of Engineering and Technology, No. 29, Xueyuan Road, Haidian District, Beijing 100083, People's Republic of China; 2 College of Architecture and Civil Engineering, Beijing University of Technology, No. 100, Pingleyuan, Chaoyang District, Beijing 100124, People's Republic of China; 3 Academy of Railway Sciences, Scientific and Technological Information Research Institute, No. 2 Daliushu Road, Haidian District, Beijing 100081, People's Republic of China

**Keywords:** integrated precast structure, lightweight recycled concrete wall with single-row reinforcement and concealed bracing, low reversed cyclic loading test, seismic resistance, seismic lines of defence

## Abstract

Given the development of precast structures for low-rise residential buildings, this study explores a new structure—namely, an integrated precast structure of lightweight recycled concrete wall with single-row reinforcement—under a lightweight steel frame filled with recycled concrete (integrated precast structure for short). The lightweight steel frame and lightweight wall cooperate to bear the forces. The applied concealed bracing, either a rebar bracing or a steel plate bracing, increases the shear resistance of the wall. The lightweight steel frame is designed to bear the vertical loading, whereas the seismic load in the horizontal direction is jointly borne by the frame and wall. This study presents the results of low reversed cyclic loading tests on nine specimens of integrated precast structures. An analysis is then carried out to investigate the mechanical properties of the specimens; based on these results, a formula for the force-bearing performance of the inclined section is developed. The results show satisfactory performance as an integrated piece; the proposed structure has two seismic lines of defence, with the lightweight wall restraint by the side frame being the first line and the steel frame being the second line. Because the failure of the wall can be categorized as shear failure, the restraint of the lightweight steel frame significantly reduces the potential damage of the wall. As the beams and columns of the steel frame tend to bend against failure, the wall filling helps resist sliding. Therefore, the reinforced joints of the connecting beams and columns show no visible signs of damage, indicating that the connection between the beams and columns is reliable. The narrow spacing of rebars and the setting of concealed bracing contribute to the increase in ductility and energy efficiency of the integrated structure and the evident reduction in the failure process. Furthermore, the recycled concrete increases the seismic resistance of the structure.

## 1. Introduction

The development of new precast structures has long been a topic of interest for researchers and scholars in the field of structural engineering and has been the subject of extensive experimental research and theoretical analysis.

Because of the global increase in steel output over the past few years, the European countries, the USA, Japan, Australia and some other countries have started to promote the application of steel structures in low-rise residences. In Sweden, which is the most industrially developed country in the world, 95% of the components of lightweight steel-structure buildings are precast. Other classic examples of precast architectural systems include lightweight steel structural systems in the USA and the residential systems—Sekisui and Toyota Homes—of Sekisui Home, Japan. The ratios of low-rise steel-frame residences to the total number of new residences are 50% in Australia, 80% in Japan, 30% in Canada and 20% in the USA [1,2].

One such structure is a precast shear wall, which has been widely applied in many countries. The precast shear wall structure in Japan generally reaches the 10th floor mark, whereas in Europe, the structure can be as high as 16–26 floors, owing to its excellent seismic resistance.

The PRESSS (Precast Seismic Structural System) project jointly initiated by the USA and Japan [3] proposed a post-tensioning prestressed precast shear wall structure with no adhesion bond. In other words, there was no connection among the rebars; rather, the post-tensioning of the prestressed ribs connected the prefabricated walls as an integrated whole. The system promised great performance in terms of seismic resistance and recoverability. Therefore, in the case of major earthquakes, the lateral displacement could be significant while the residual deformation could be neglected. This was because damage mainly progressed from the connection of the prefabricated members, which could be easily restored afterwards. Pavese & Bournas [4] performed seismic resistance tests on a full-scale precast slab-type shear wall structure and a full-scale two-storey H-shaped precast shear wall structure. The results indicated that the failure of the specimens could be categorized as bending-shear failure, and thanks to the limited degradations in strength and stiffness, the specimens met the standards of seismic resistance.

Mehmet & Tugrul [5] attempted to improve a concrete frame structure by introducing precast high-strength concrete walls, which significantly enhanced the strength and stiffness of the frame. Brian & Yahya [6] used mild steel and high-strength unbonded post-tensioning steel to improve the sliding resistance of the horizontal joints of a precast concrete (PC) shear wall. Park *et al*. [7] carried out a dynamic analysis on a 15-storey building with precast composite frames, where the PC columns and beams were joined through a simple connection, leading to the identification of the dynamic performance of the structure. Jia *et al*. [8,9] studied the seismic performance of the precast structure of a frame-supported multi-ribbed composite wall with a large space at the bottom under cyclic loading. The results showed that the structure as a whole provided extraordinary seismic performance, because of the unique layer-by-layer embedded design of the wall.

Pavese & Bournas [4] experimentally investigated the behaviour of prefabricated reinforced concrete sandwich panels under simulated seismic loading through a large experimental campaign. The results indicated that the prefabricated walls of the structural system investigated herein seem to meet all requirements of Eurocode 8 for walls to be designed as ‘large lightly reinforced walls'. Wu *et al*. [10] proposed a quasi-static and dynamic cyclic lateral loading test on a type of precast slender composite shear wall system. In this new precast structural wall system, concrete-filled steel tubes (CFSTs) were used to entirely replace the longitudinal reinforcement in the boundary elements of conventional reinforced concrete shear walls. At joints, the CFSTs and wall web reinforcement were connected by sleeves filled with high-strength mortar. The results indicated that the use of CFSTs increased the lateral strength and deformation capacity, and the effectiveness of the proposed detailing of sleeve-mortar connections in load transfer was validated. Lim *et al*. [11] developed an innovative PC T-wall panel system; to confirm its lateral-load-resisting and seismic performance, reversed cyclic tests of two two-thirds scale PC T-walls with and without diagonal reinforcing bars were conducted under displacement control. The test results showed that the T-wall specimen without diagonal reinforcement performed reasonably well in terms of lateral stiffness, strength and ductility. A full-scale three-storey precast building was subjected to a series of pseudo-dynamic (PsD) tests in the European Laboratory for Structural Assessment (ELSA). An innovative connection system, embedded in the precast elements, was then activated to create emulative beam–column connections. The PsD test results showed that, when activated at all floors, the proposed connection system was quite effective as a means of implementing dry precast emulative moment-resisting frames [12].

Though researchers and scholars in the field have investigated the performance of the members and joints of precast structures, very few studies have been conducted on the force-bearing performance of the precast frame and wall as a whole, and even fewer studies have been conducted on the performance of an integrated precast structure of lightweight recycled concrete wall with single-row reinforcement, under a lightweight steel frame filled with recycled concrete (integrated precast structure for short).

In addition, the application of recycled concrete helps the industry properly resolve the pollution challenges posed by concrete waste. At the same time, it reduces the consumption of natural resources and the construction costs, thereby contributing to the strategy of sustainable development of building resources. Much work has been carried out to study the structure and members of recycled concrete structures, which has helped clarify the basic properties of these materials.

Chen *et al*. [13] studied the performance of a fully recycled coarse concrete shear wall with a shear span ratio of 2, and the results indicated that apart from fairly good performance in elastic deformation, the structure's performance was similar to that of a normal concrete structure. Zhang *et al*. [14] conducted vibration tests on a recycled concrete shear wall with concealed bracing, which had a shear span ratio of 1.5. It was found that at any stage, the measured displacement response of the specimens, particularly that of the medium and high ones, was much lower than that of recycled concrete shear walls in general. In other words, there was improvement in the seismic resistance of the specimens.

Currently, very few studies in the field of recycled concrete structures have targeted the commonly found low-rise residential buildings.

Considering the development of precast low-rise residential buildings, this study explores a new structure, the integrated precast structure. The concealed bracing applied—either rebar bracing or steel plate bracing—increases the shear resistance of the wall. For the lightweight frame, the lightweight steel pipe beams/columns—both filled with recycled concrete—are connected by bolts at the rib-reinforced joints, thereby significantly improving the strength and stiffness of the joint area. The lightweight wall has bar-type steel plates around it for welding to the rebars. Because the bar-type steel plates of the lightweight wall and the steel lathings of the beams and columns are connected by bolts, a force-bearing system is developed, in which the lightweight steel frame and lightweight wall cooperate in bearing the forces. It is noted that the lightweight steel frame is mainly designed to withstand the vertical loading, whereas the lightweight wall withstands the horizontal loading. The sliding resistance is jointly developed by the frame and wall; therefore, the structure as a whole possesses satisfactory seismic resistance.

The integrated precast structure can be arranged as a shear wall unit, embedded to the frame structure or shear wall structure.

Compared with the existing structure of precast steel frame, the newly proposed structure has some new features that satisfy the demand for the industrialization of precast low-rise residential buildings and the need to recycle concrete waste: first, the lightweight steel pipe beams and columns filled with recycled concrete; second, the reinforced joint of the beams and columns; and third, the connection of the lightweight steel frame and lightweight recycled concrete wall with single-row reinforcement and concealed bracing, which ensures that the frame and wall jointly bear the force imposed.

For the structure proposed in this study, the first seismic line of defence is the lightweight recycled concrete shear wall restrained by the lightweight steel pipe frame filled with recycled concrete; in other words, the frame and the wall work together in developing seismic defence. During failure of the wall, the second seismic line of defence, the lightweight steel frame, becomes important.

This study carries out low reversed cyclic loading tests on the integrated precast structure and investigates the effect of rib spacing and the setting of oblique concealed bracing on the failure process, hysteretic performance, load-bearing performance, ductility, stiffness and energy consumption.

## 2. Overview of experiments

### 2.1. Experimental procedure

In this study, nine pieces of integrated precast structures were designed. Some of the lightweight walls were embedded with either rebar bracing or steel plate bracing (see table 1 for the specifications).

**Table 1.** Specifications of the integrated structure of precast lightweight steel frame–lightweight wall.

group no.	specimen no.	thickness of the wall panel	rebar spacing (mm)	reinforcement ratio (%)
1	B1Z1-60-1	60 mm	100	0.33
	B1Z1-60-2	60 mm + concealed rebar bracing	100	0.33
	B1Z1-60-3	60 mm + concealed plate-type bracing	100	0.33
2	B2Z1-60-1	60 mm	150	0.22
	B2Z1-60-2	60 mm + concealed rebar bracing	150	0.22
	B2Z1-60-3	60 mm + concealed plate-type bracing	150	0.22
3	B3Z1-60-1	60 mm	200	0.16
	B3Z1-60-2	60 mm + concealed rebar bracing	200	0.16
	B3Z1-60-3	60 mm + concealed plate-type bracing	200	0.16

Steel pipe columns and beams filled with recycled concrete were connected via reinforced joints, which then constituted the lightweight frame. I-beams, with a length of 400 mm, were used as the foundation of the specimens. The beams and columns of the lightweight frame were made of square-shaped steel pipes, with a side length of 100 mm and a thickness of 4 mm, filled with recycled concrete. The steel lathings, with a thickness of 4 mm and a height of 40 mm, were welded to the frame columns and frame beams for connection to the wall. In other words, the lathings on the columns and the beams were bolted to the bar-type steel plates of the recycled concrete wall with single-row reinforcement. A spacing of 20 mm was reserved between the foundation beam and the steel pipe beam at the bottom to ensure that the deformation of the steel pipe beam was not subjected to restraint of the foundation beam. It should be noted that the column base and foundation beams were connected via M20 high-strength bolts.

**Figure 1.**
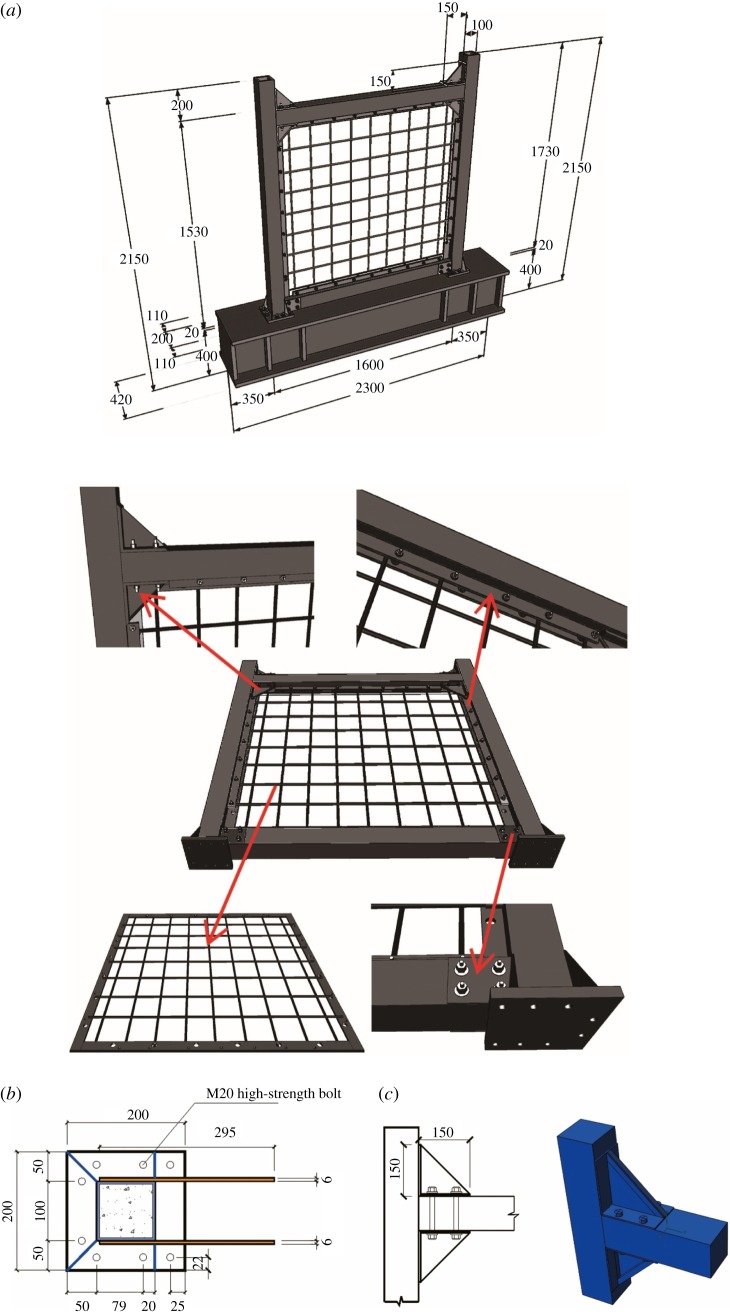
Three-dimensional dimensions and structures of the specimens: (*a*) dimensions and construction, (*b*) column base and (*c*) beam–column joint.

The beam–column connection applied to the steel frame was a reinforced joint (dual-L-shaped joint with oblique ribs) composed of an equilateral steel angle with two plate-type stiffening ribs having a triangular shape, the hypotenuse of which was welded to 20 mm diameter steel bars for pressure and instability resistance. The vertical plane of the steel angle was welded to the column at the side, and the horizontal plane was bolted to the beam. Figure 1 shows the dimensions and structure of the specimen.

The steel skeleton of the wall was composed of 4 mm thick and 40 mm wide frame plates, 5 mm diameter rebars for both directions, and concealed bracing—either rebar bracing or plate-type bracing. In the steel skeleton, the concealed bracings were arranged at both sides of the single-row reinforcement in a criss-crossed fashion and then fastened by 1 mm diameter steel wires. It should be noted that bracing could also be welded to the reinforcement for engineering application. The cross-section of the plate-type bracing—40 mm in height and 4 mm in thickness—was identical to that of the rebar bracing—10 mm in diameter—in terms of total area. The frame plate of the wall with bolt holes was connected to the steel lathings of the beams and columns via M10 bolts. The arranged rebars had three reinforcement ratios of 0.33, 0.22 and 0.16%. The recycled concrete filled into the steel pipe was similar to that applied in the wall, both being recycled coarse concrete. The coarse aggregate had a 100% replacement ratio and 5–10 mm grain diameter. The fine aggregate selected was natural sand, and the designed strength of the concrete was C40. Furthermore, this study used arranged high-strength rebars for the wall; Q235 rolled steel for the plate frame, steel lathing of the beams and columns, and plate-type bracing; and seamless steel pipe for the square pipe.

### 2.2. Fabrication of structure and material performance

**Figure 2.**
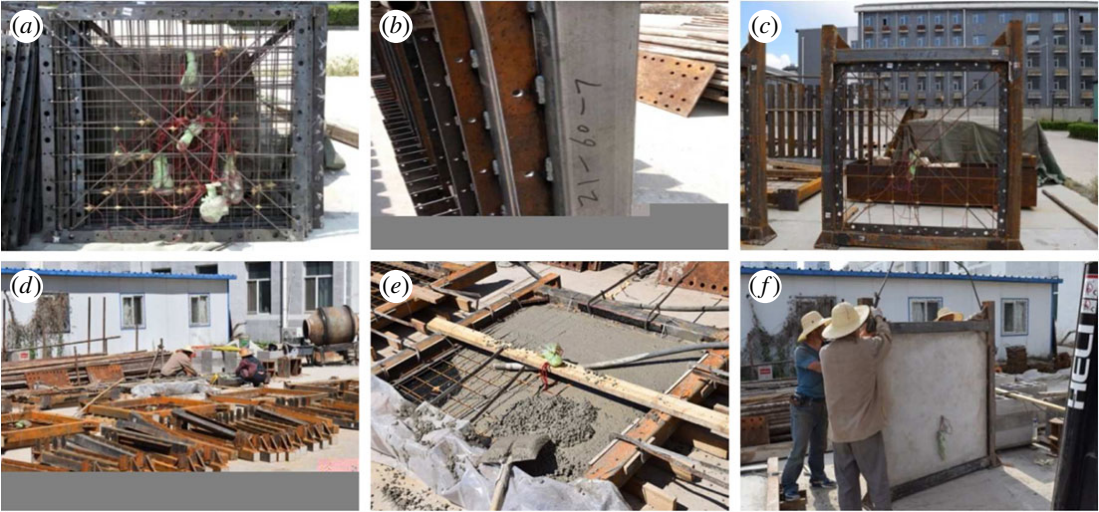
Fabrication of the members and joints of the precast specimens: (*a*) steel skeleton of the lightweight wall with rebar bracing, (*b*) steel lathing welded to the steel pipe column, (*c*) precast lightweight steel frame and steel skeleton of the lightweight wall, (*d*) steel pipe columns filled with recycled concrete, (*e*) horizontal pouring of recycled concrete for the fabricating of the lightweight wall and (*f*) specimen hoisting after curing.

As a first step of preparing the specimens, lightweight steel pipe columns and beams filled with recycled concrete were manufactured. Reinforced joints were then made and welded to the corresponding locations on the frame columns. The construction of the frame was completed as the columns and beams were fully connected. The steel skeleton of the lightweight wall was then built and connected to the frame. After pouring the recycled concrete into the skeleton, the specimen was ready. Figure 2 shows the fabrication of the specimens and the fabrication of the members and joints. When the factory-made walls had consistent dimensions and a smooth and neat appearance, the lightweight skeleton and steel frame were bolted before casting of the concrete to avoid any damage to the wall during manufacturing of the specimen. This alternative approach ensured consistent force-bearing performance in all specimens.

The recycled concrete filled inside the steel pipe—identical to the concrete of the wall—was made of recycled coarse aggregate with a grain size of 5–10 mm (replacement rate 100%), natural sand, ordinary Portland cement, water, mineral powder, fly ash and water reducer. All ingredients were mixed at a pre-defined ratio in a mixer. Table 2 presents the mechanical performance of the recycled concrete measured on site, and table 3 indicates the mechanical performance of the steel members.

**Table 2.** Mechanical performance of the recycled coarse concrete.

designed grade	components cement : water : fine aggregate : coarse : fly ash : fine ore	compressive strength *f*_cu_ (MPa)	elastic modulus *E*_c_ (MPa)
C40	1 : 0.49 : 2.28 : 2.28 : 0.21 : 0.21	41.15	3.15 × 10^4^

**Table 3.** Mechanical performance of the rebar and the steel pipe measured on site.

steel type	steel specification (mm)	yield strength *F*_y_ (MPa)	strength limit *F*_u_ (MPa)	elongation *δ* (%)	elastic modulus *E* (MPa)
rebar/rebar bracing	*Φ*5	680	786	5.5	2.09 × 10^5^
steel plates/steel lathings/steel plate bracing	40 × 4	309	467	25.27	2.11 × 10^5^
square pipe	100 × 100 × 4	375	477	23.23	2.18 × 10^5^

### 2.3. Loading scheme

**Figure 3.**
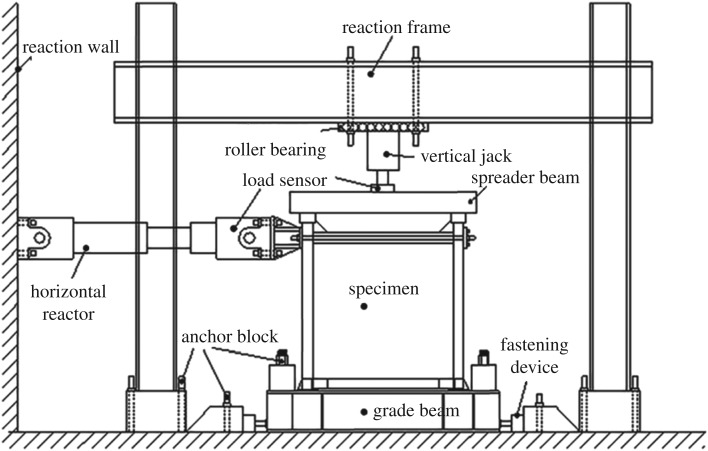
Diagrammatic sketch of the loading device.

**Figure 4.**
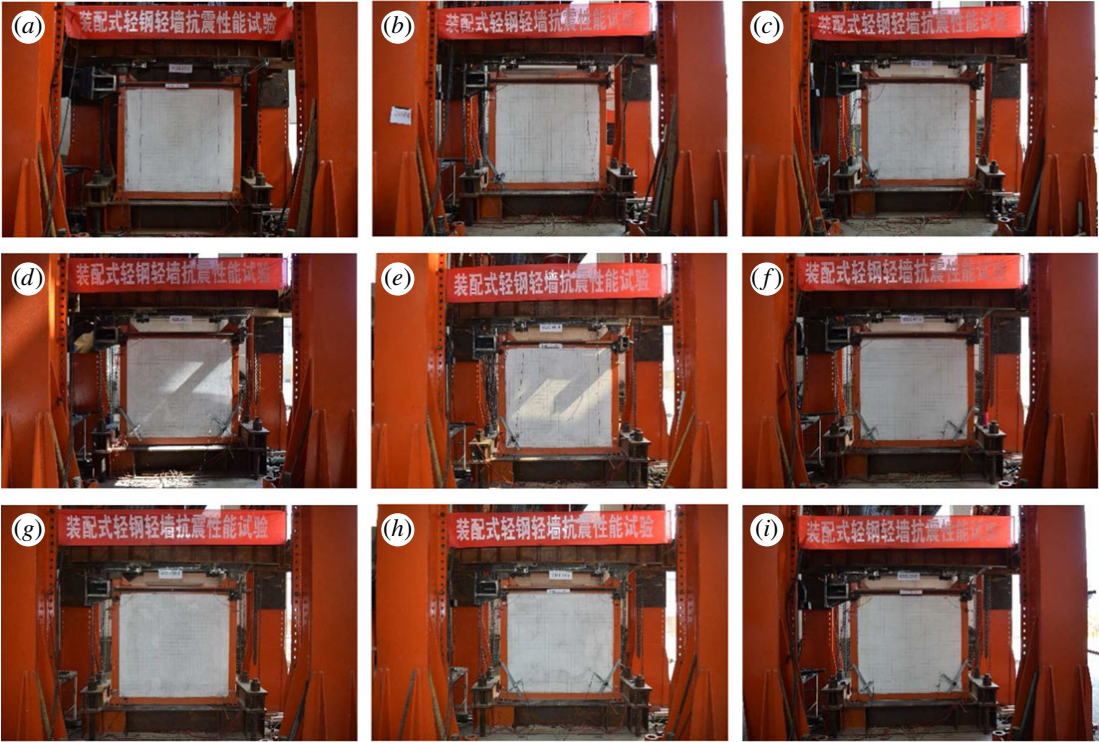
Loading site of the specimens: (*a*) B1Z1-60-1, (*b*) B1Z1-60-2, (*c*) B1Z1-60-3, (*d*) B2Z1-60-1, (*e*) B2Z1-60-2, (*f*) B2Z1-60-3, (*g*) B3Z1-60-1, (*h*) B3Z1-60-2 and (*i*) B3Z1-60-3.

Figure 3 shows the loading device used. As the experiment started, a 600 kN load was first applied to the centre of the top surface of the spreader beam. The vertical loadings, on the other hand, were symmetrically imposed on the top of the side columns of the steel frame via the spreader beam, the loading value of which was maintained throughout the test. The axial compressive ratio of the column—0.35 to be exact—was determined in accordance with the standard value of the material strength. Therefore, it represented the experimental ratio. If the calculation was carried out in the light of the designed value, the axial compressive ratio would stand at 0.59. Low reversed cyclic loadings were then horizontally imposed to the axis of the frame beams, with the loading point being 1480 mm from the top surface of the foundation. Figure 4 shows the loading site of the specimens.

**Figure 5.**
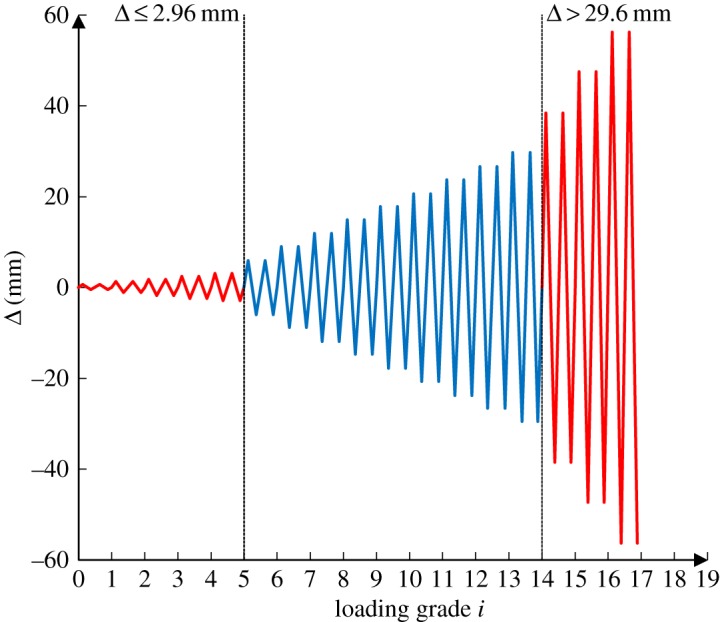
Loading scheme.

The loading scheme is shown in figure 5. The loading scheme with the displacement control was applied as follows: before the displacement angle reached 1/500 and 1/50, the angle increments were at 1/2500 and 1/500, respectively. As the displacement angle exceeded 1/50, the increment value was raised to 3/500. At each grade of the loading scheme, the loading cycle was then repeated once until the experiment ended, at which point, the specimen failed, it was impossible to continue the loading, or the horizontal loading dropped to less than 85% of the peak value. According to the *Code for Seismic Design of Buildings* (GB50011-2010 [15]), for the concrete frame, the displacement angle incurred during the elastic deformation reached no higher than 1/500, and that induced during the elastic–plastic deformation was 1/50. In the middle of the experiment, the loading was increased at a consistent rate. Because the jack exerted a pushing force, the direction of the horizontal force was defined as the positive direction.

**Figure 6.**
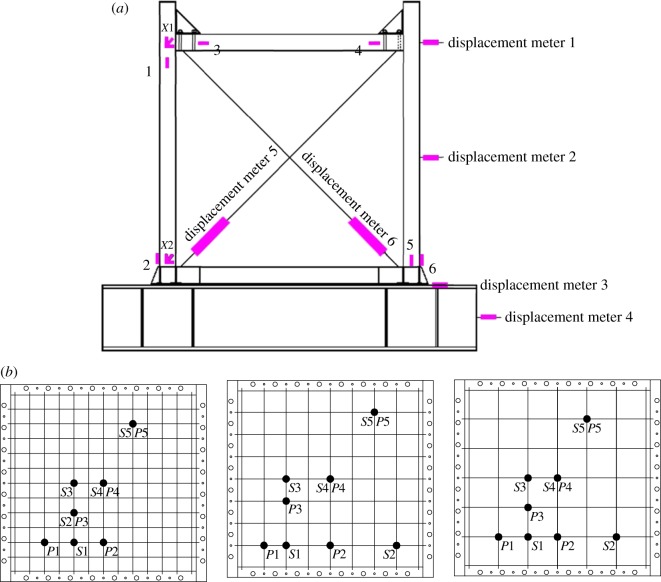
Arrangement of the measurement points: (*a*) outer frame and (*b*) steel skeleton of the wall.

The measurement points were set as follows: force sensors were arranged on the top of the vertical and horizontal jacks. Displacement meters nos. 1 and 2 were for the displacement of loading points; nos. 3 and 4, sliding base; nos. 5 and 6, shear deformation of walls; *X*1 and *X*2 are strain rosettes and nos. 1–6 are the vertical strain gauges. Measurement points *P*1–5 and *S*1–5 are set for the strains of horizontal and vertical reinforcements, respectively (figure 6).

Regarding data collection, the IMP data collection system automatically collected the data on loading, displacement and strain in real time. At the same time, any crack on the recycled concrete wall or any buckle on the steel frame was observed manually with the experimental phenomenon being properly recorded.

## 3. Results and analysis

### 3.1. Test results

Take B1Z1-60-1, B2Z1-60-2 and B3Z1-60-3, for example.

#### 3.1.1. B1Z1-60-1

When the displacement angle reached 1/830, vertical cracks were first observed at the lower joint of the wall and steel pipe columns, which were then widened and extended as the loading continued. At that point, minor cracks started to appear at the interface of the wall and steel pipe columns, and a slight dislocation was observed. At the displacement angle of 1/515, cross-cracks appeared along the principal diagonal of the wall accompanied by a cracking noise, and at 1/450, many cracks visible to the naked eye appeared across the wall. When the displacement angle increased to 1/163 under increased loading, the existing cracks continued to extend at both ends, leading to the occurrence of continuous cracks. It was noted that cross-cracks parallel to the principal diagonal started to be visible in large numbers. At the displacement angle of 1/96, the recycled concrete at the corner of the wall started to spall off, with the cracks further widened to 3 mm. Meanwhile, as the wall and steel columns cracked further apart and the bolt connections between the steel lathing and steel plates were loosened, visible dislocation of the wall and steel columns was noted. As the loading continued to increase, the displacement angle increased to 1/79. At that point, the concrete continued to spall off until the concrete at the corner of the wall was finally crushed.

**Figure 7.**
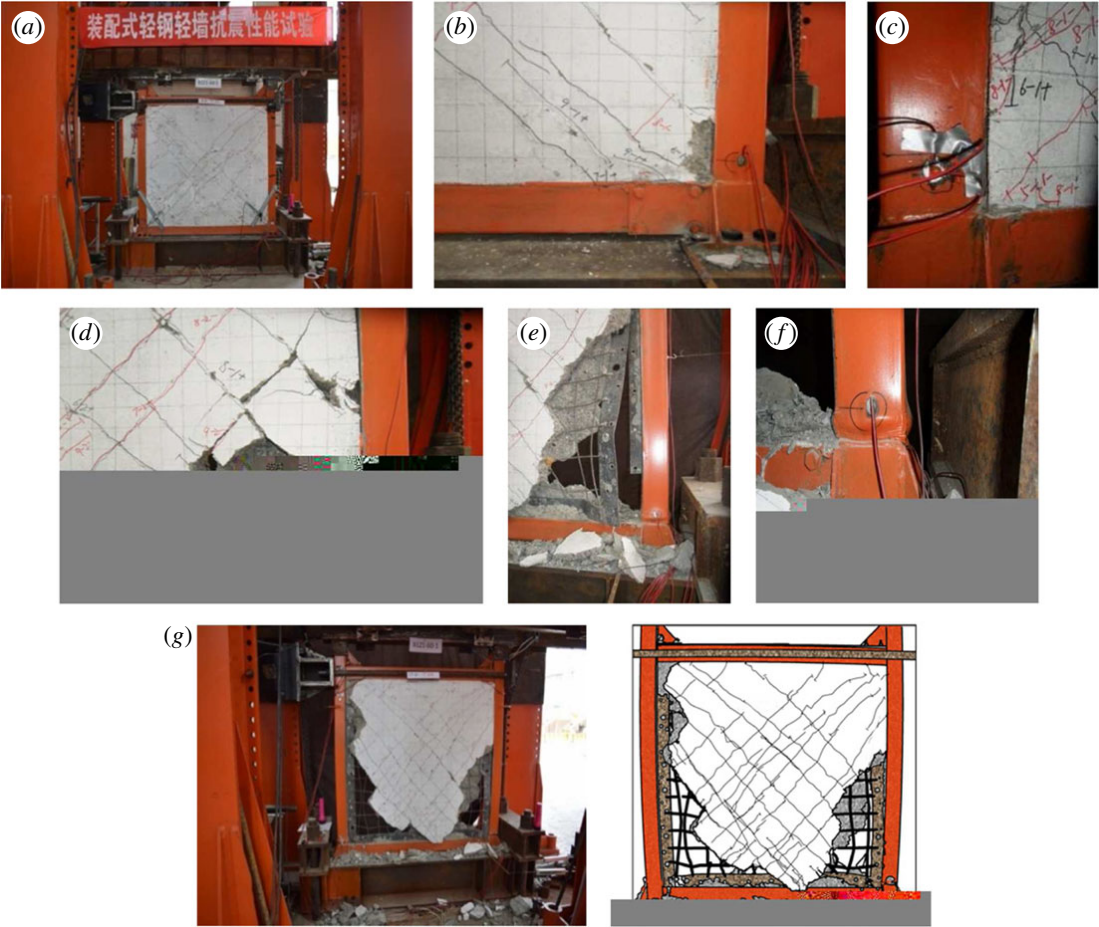
Partial and overall damage observed in specimen B1Z1-60-1: (*a*) cracks are evenly distributed on the wall, (*b*) recycled concrete at the bottom corner of the wall starts to spall off under the loading, (*c*) the vertical crack between the wall and steel pipe column is widened and starts to extend at both ends, (*d*) recycled concrete at the lower wall continues to spall off, (*e*) the steel plates—detached from the steel lathing—yield, and the embedded rebars deform, (*f*) both the inner and outer sides of the steel columns start to buckle with the latter more visible than the former and (*g*) overall damage.

A 2 mm buckle was observed at the outer surface of the bottom part of the steel column. When the angle increased to 1/36, the joints of the wall corner and steel columns were severely damaged with the rebars partially exposed. The experiment was terminated as the angle reached 1/26. In this study, it was observed that the wall rebars, detached from the steel plates, were deformed or even broken, the lower right corner of the steel plates started to curl up, and the bottom part of the column buckled severely. Figure 7 shows the partial and overall damage in the specimen.

#### 3.1.2. B2Z1-60-2

**Figure 8.**
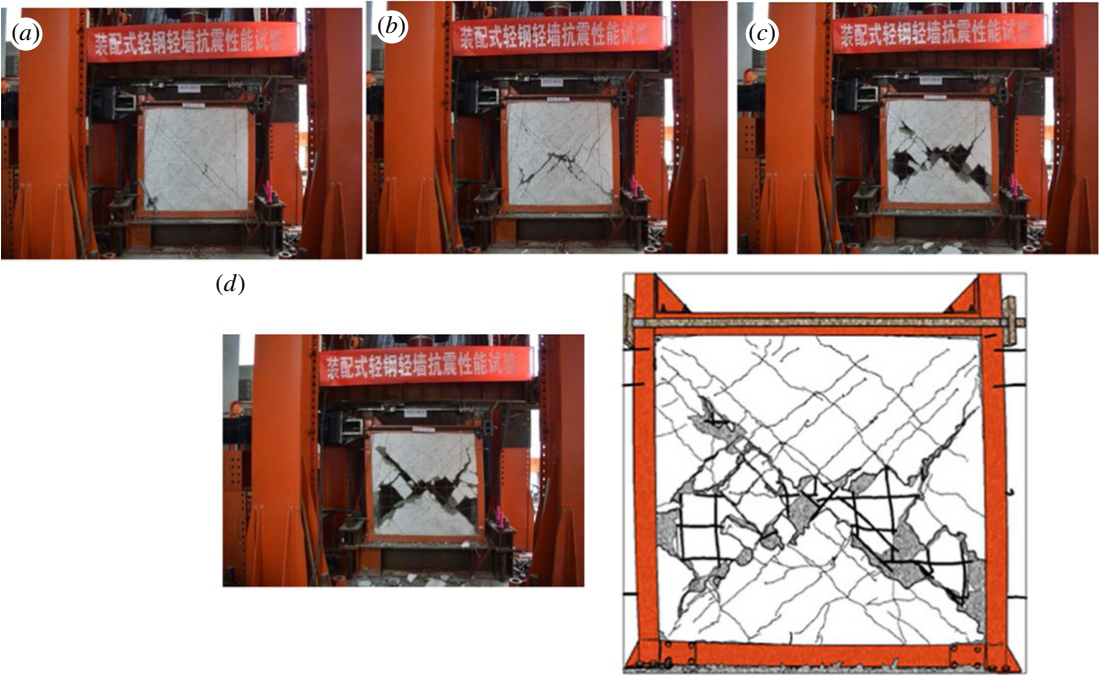
Partial and overall damage in specimen B2Z1-60-2: (*a*) major oblique cracks are widened, (*b*) the recycled concrete in the central area of the wall starts to spall off, (*c*) the spalling spreads out from the central area to the principal diagonal of the wall and (*d*) overall damage.

When the displacement angle reached 1/710, cross-cracks started to appear at the diagonal area of the wall. As the loading continued, the displacement angle increased to 1/515, and the cross-cracks grew in number, spread out across the wall. As the loading further raised the angle to 1/96, concrete spalling took place at the central areas of the wall. At 1/68, the cracks at the intersection of the rebar bracing grew in depth accompanied by deterioration in concrete spalling. As the displacement increased, the spalling of concrete developed from the middle to the side of the wall. At an angle of 1/19, the experiment was stopped. It was observed that despite the slight bending of the steel columns, the specimen still showed fairly good performance in terms of collapse resistance, as the connection between the wall and steel columns was sound. Figure 8 shows the partial and overall damage that occurred in the specimen.

#### 3.1.3. B3Z1-60-3

**Figure 9.**
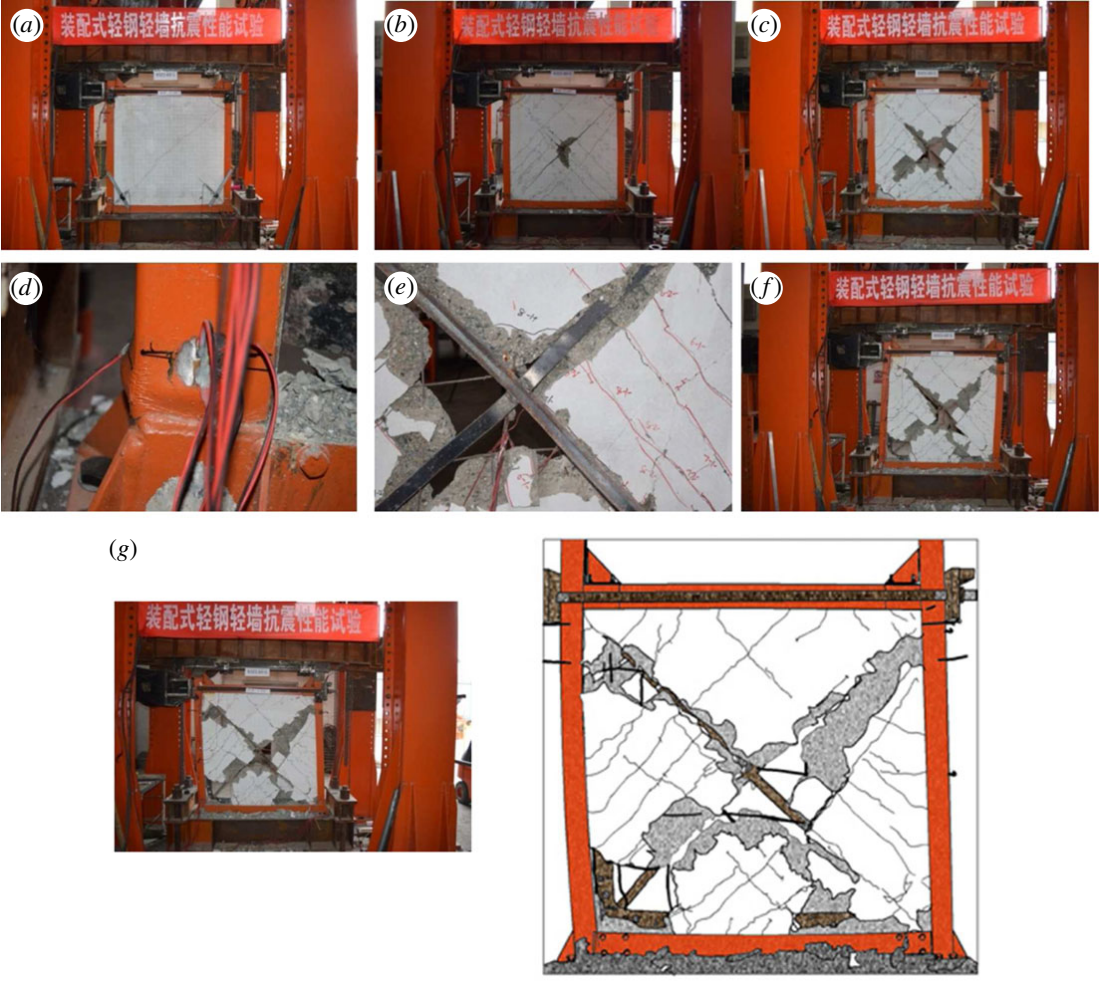
Partial and overall damage in specimen B3Z1-60-3: (*a*) the outer surface of the steel columns buckles, (*b*) the recycled concrete at the intersection of the major oblique cracks spalls off, (*c*) the recycled concrete spalling spreads out, (*d*) the outer surface of the steel columns buckles, (*e*) the steel plate bracing buckles, (*f*) the recycled concrete along the steel plate bracing suffers from severe spalling as the steel plate at the lower left corner bends and (*g*) overall damage.

Two major oblique cracks appeared as the displacement angles reached 1/745 and 1/500. As the loading continued, the angle increased to 1/425, where cracks parallel to the major cracks grew in number. Afterwards, the major cracks were deepened at the displacement angle of 1/119. At 1/92, the recycled concrete at the intersection of the major oblique cracks started to spall off, which then spread out along the direction of the steel plate bracing to the edges of the specimen as the loading continued. At that point, the wall was divided into four triangular walls, all of which exhibited fairly good performance in terms of stability. As the displacement angle increased to 1/29, the steel plate at the corner areas started to bend, which then resulted in its detachment from the steel columns. The experiment stopped at the displacement angle of 1/25. By that time, the steel columns buckled slightly with signs of tear, and the embedded rebars were deformed and some were broken. However, the concealed bracing remained in good shape, and the connection between the wall and the steel columns remained sound. Figure 9 shows the partial and overall damage in the specimen.

The test results of the three groups of specimens showed that the new design ensured a fairly good connection between the precast lightweight steel frame and the thin recycled concrete wall, which was enhanced by the embedded rebar bracing. In the cases where the spacing of the wall rebars was 150 and 200 mm, the spalling of recycled concrete started from the centre and then spread out along the principal diagonal to the outer range of the wall. However, when the spacing was reduced to 100 mm, the spalling occurred at the interfaces of the wall and steel columns. Upon failure of the specimens, the steel plates around the wall curled up, but the bolt connections remained sound with no signs of breakage, and the overall integrity of the specimens was satisfactory. Because of the rib-reinforced joints connecting the lightweight steel pipe beams and columns (filled with recycled concrete), the specimens exhibited better performance in bending resistance and distortion resistance at the beam–column joint.

In general, the structure represents both a force-bearing system and functional system. It is a force-bearing system, because the thin recycled concrete wall withstands the shear force, while the lightweight steel frame bears the vertical loading, with the two showing fairly good integrity. It is a functional system, because the system has two distinct seismic lines of defence—namely, the thin recycled concrete wall and the lightweight steel frame. After failure of the first line of defence, the steel skeleton and the lightweight frame jointly withstand the loading imposed.

### 3.2. Hysteretic performance

**Figure 10.**
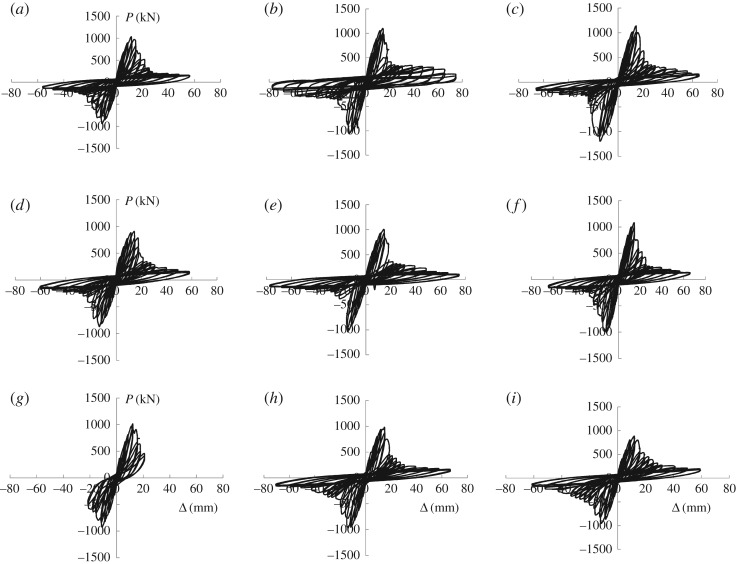
Hysteretic curves: (*a*) B1Z1-60-1, (*b*) B1Z1-60-2, (*c*) B1Z1-60-3, (*d*) B2Z1-60-1, (*e*) B2Z1-60-2, (*f*) B2Z1-60-3, (*g*) B3Z1-60-1, (*h*) B3Z1-60-2 and (*i*) B3Z1-60-3.

Figure 10 shows the hysteretic curves of loading *P* (kN) and displacement Δ (mm) for all nine specimens.

It can be observed in figure 10 that given the butterfly shape of the hysteretic curves obtained, the structure displays two distinct seismic lines of defence: the lightweight recycled concrete wall with single-row reinforcement and the lightweight steel pipe frame filled with recycled concrete. As the lightweight recycled concrete wall with single-row reinforcement gradually phases out its role of force bearing, the hysteretic curves descend rapidly. As the curve reaches the lower limit of force bearing, the first line of defence failed and the second line—the lightweight steel frame—worked with the concealed bracing—either rebar bracing or steel plate bracing—forming a truss system of force bearing. In other words, the setting of the bracing leads to fuller hysteretic curves. Because the bond strength between rebars and recycled concrete exceeds that between steel plates and recycled concrete, it can be concluded that the rebar bracing outshines its counterpart in energy consumption, making it a preferable design. It should be noted that smaller spacing between the arranged rebars translates into fuller hysteretic curves and higher energy consumption.

### 3.3. Analysis of force-bearing performance and ductility

**Figure 11.**
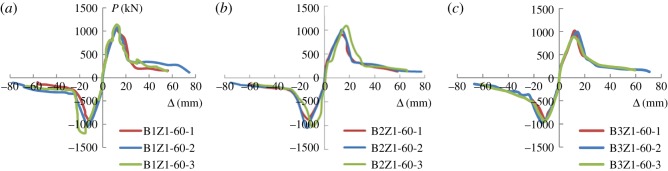
Skeleton curves: (*a*) group 1, (*b*) group 2 and (*c*) group 3.

For the experiments, the characteristic points are selected according to the following rules. The cracking status refers to the loading and displacement observed as more minor cracks start to appear on the wall. The peak status and failure status are determined based on the data collected, and the yielding status is calculated using the energy equivalence method. Figure 11 shows the skeleton curves of loading *P* (kN) and displacement Δ (mm).

It can be observed in figure 11 that the skeleton curves of all three groups of specimens go through the elastic stage, elastic–plastic stage and decreasing stage. At the first stage, or the elastic stage, the skeleton curves of all specimens show a similar pattern of increase with a relatively large slope, implying that the primary stiffness of the structure is high. In addition, the concealed bracing—rebar type or steel plate type—increases the force-bearing performance and ductility of the structure. Compared with its counterparts, the new structure shows a greater displacement angle upon failure as well as a better collapse resistance. It is noted that the oblique rebar bracing has been proved to be a more viable design than the plate-type bracing. It is also worth noting that because of the wide spacing between the rebars, specimen B3Z1-60-1 (with 200 mm spacing) fails to display the two lines of defence. Because the wall yields at an early stage, it cannot share the loading imposed on the lightweight frame. This explains why the displacement at failure largely falls short compared with the other specimens.

Table 4 presents the main characteristic points of the specimens. For the experiments, the characteristic points are selected according to the following rules.

**Table 4.** Main characteristic points of the specimens.

	cracking		yielding		peak		failure			ductility ratio
specimen no.	Δ_cr_ (mm)	*F*_cr_ (kN)	Δ_y_ (mm)	*F*_y_ (kN)	Δ_m_ (mm)	*F*_m_ (kN)	Δ_d_ (mm)	*F*_d_ (kN)	*θ*	*μ*
B1Z1-60-1	3.09	414.65	7.14	825.16	11.23	986.29	56.21	125.7	1/26	1.85
B1Z1-60-2	5.24	586.89	8.54	912.58	13.78	1076.78	76.13	110.58	1/20	2.18
B1Z1-60-3	3.16	376.32	9.75	980.16	14.56	1166.75	65.64	133.39	1/22	1.61
B2Z1-60-1	1.95	294.69	8.59	783.93	13.83	889.53	59.26	130.66	1/26	1.87
B2Z1-60-2	2.99	346.45	10.06	861.58	14.43	1023.84	76.22	115.01	1/20	1.94
B2Z1-60-3	3.05	397.05	8.10	881.41	12.54	1045.74	65.2	125.83	1/22	1.67
B3Z1-60-1	0.76	155.69	8.4	796.85	11.62	969.98	21.27	461.07	1/68	1.54
B3Z1-60-2	3.03	366.53	8.57	823.45	14.3	973.77	68.66	132.35	1/22	1.89
B3Z1-60-3	3.39	375.4	7.15	775.26	11.8	918.35	60.33	151.91	1/26	1.78

The cracking status refers to the loading and displacement observed as more minor cracks start to appear on the wall. The peak status and failure status are determined based on the data collected, and the yielding status is calculated using the energy equivalence method.

The paper takes reference from the rules on displacement ductility ratio specified in the *Specification of Test Methods for Earthquake Resistant Buildings* (JGJ101-1996 [16]) to study the ductility performance of the specimens. To be more specific, the displacement ductility ratio is as follows:

3.1 $$\mu =\frac{\Delta_{u}}{\Delta_{y}},$$

where the elastic–plastic displacement of the specimens Δ*_u_* represents the displacement as the horizontal loading drops to 85% of the peak values (*F*_m_), and Δ_y_ is the yield displacement.

It can be observed in table 4 that all specimens in the peak state register a displacement angle of 1/112. Because the topic of this study falls in the category of frame–shear wall structures, the displacement angle between the elastic–plastic layers has a maximal value of 1/100 according to the *Code for Seismic Design of Buildings* (GB50011-2010 [15]). Therefore, during a major earthquake, the seismic load imposed stands on par with the force-bearing limit of the integrated structure proposed. In other words, prior to failure of the first seismic line of defence, the force-bearing capacity of the structure has a major role to play when an earthquake occurs. Afterwards, upon failure of the first seismic line, the wall is still capable, to some extent, of resisting the effect of the loading imposed, because its failure consumes some of the seismic energy. At the later stage of the experiment when the wall fails, the second line of defence, the lightweight steel frame, acts. It is noted that the average displacement angle at failure of the specimen is 1/24, which shows that the structure exhibits fairly good performance in terms of collapse resistance and ductility.

The rebar bracing elevates the overall performance of the structure, which then translates into better ductility. On the other hand, given the limited bond strength of the plate-type bracing and the recycled concrete, the plate-type bracing undermines the deformation performance of the structure. It should be noted that the decrease in the reinforcement spacing leads to a surge in crack loading, yield loading and loading peak, whereas the widening of the spacing most affects crack loading, because the smaller spacing of arranged reinforcement results in closer and more evenly distributed cracks, the width of which is evidently smaller. Exceedingly wide spacing can undermine the integrity and ductility of the overall specimens.

### 3.4. Stiffness degradation

Secant stiffness *K_i_* of the specimen is calculated as follows:

3.2 $$K_{i}=\frac{\left|F_{i}^{+}|+|F_{i}^{-}\right|}{\left|\Delta_{i}^{+}|+|\Delta_{i}^{-}\right|},$$

where *i* is the cyclic progression; *K_i_* is the secant stiffness at cyclic progression *i*; *F_i_* is the loading peak at cyclic progression *i*; Δ*_i_* is the displacement at cyclic progression *i*; + or – is the positive or negative direction of horizontal force, respectively.

**Figure 12.**
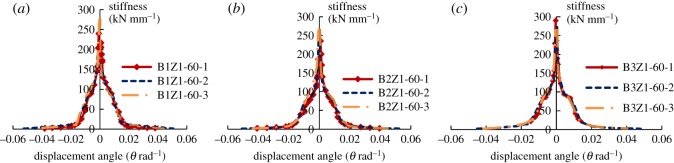
Curves for stiffness degradation: (*a*) group 1, (*b*) group 2 and (*c*) group 3.

Figure 12 shows the relationship between the stiffness degradation and displacement angle for the three groups of specimens.

In figure 12, it can be observed that the stiffness degradation of all three groups of specimens follows a similar pattern, which can be divided into three stages. At the first stage, the wall, which has a fairly high primary stiffness (250–300 kN mm^−1^), mainly bears the lateral force. As the displacement angle increases, visible cracks are observed on the wall. The increase in cracks causes a sudden reduction in the lateral stiffness, which then continues until failure of the first seismic line of defence, the thin recycled concrete wall. At the second stage of stiffness degradation, as the displacement angle increases, the lightweight steel frame mainly bears the effect of loading. At this time, the second line of defence acts, during which the stiffness degradation decreases. At the third stage, the lightweight steel frame cooperates with the rebar skeleton in bearing the force, leading to stabilization of the stiffness degradation. Because of the consistent curves of stiffness degradation, the oblique bracing has an insignificant role in undermining the deterioration. It is also observed that the increase in the spacing between rebars leads to a more stable degradation of stiffness.

### 3.5. Analysis of the strength degradation

This study introduces the coefficient of overall loading degradation *λ_i_* to describe the overall degradation of the specimens throughout the loading process (see the below equation).

3.3 $$\lambda_{i}=\frac{P_{i}}{P_{\max}},$$

where *P_i_* is the peak value within loading grade *i*, and *P*_max_ is the highest loading peak throughout the process.

**Figure 13.**
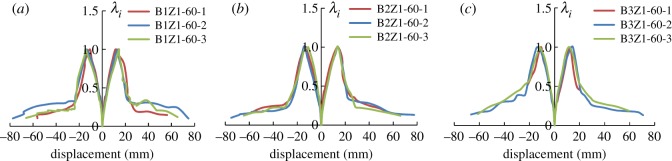
Coefficient of overall loading degradation subjected to displacement changes: (*a*) group 1, (*b*) group 2 and (*c*) group 3.

Figure 13 shows the changes in the coefficient of overall loading degradation as the displacement changes.

It can be observed in figure 13 that the strength degradation of all three groups of specimens is similar. At the initial stage, *λ_i_* increases with increasing displacement, and as the curve reaches the peak, *λ_i_* starts to decrease. It is noted that the strength of the third group of specimens (with 200 mm rebar spacing) degrades more rapidly than the other two groups. It is also observed that the stiffness of the three groups deteriorates in a more stable manner after the initiation of the second seismic line of defence than it does before. This means that the lightweight steel frame and the wall skeleton cooperate in the task of force bearing, which leads to a stable decrease in the force-bearing capacity. During this process, the oblique bracing also contributes by helping reduce the deterioration of the force-bearing capacity. During failure of all three groups of specimens, *λ_i_* varies between 0.1 and 0.19. From figures 7 to 9, it can be concluded that because of the well-structured connection between the lightweight steel frame and the recycled concrete wall, the specimen shows satisfactory performance in collapse resistance despite the reduction in force-bearing capacity.

### 3.6. Energy consumption of each seismic line of defence

The total area of the hysteretic curves is introduced to measure the energy absorption of the specimen subject to repeated seismic influence, and the accumulated energy consumption is calculated by adding the area of each hysteretic curve obtained at the characteristic points amid and prior to cyclic loading.

Table 5 lists the energy consumption at each stage and each seismic line of defence for all nine specimens and lightweight recycled concrete-filled frames of identical dimensions manufactured by the research team.

**Table 5.** Energy consumption at each stage.

specimen no.	yielding	peak	failure	first seismic line of defence	second seismic line of defence
B1Z1-60-1	14529.50	26007.79	174563.33	87637.39	129653.2
B1Z1-60-2	22546.17	16975.48	328065.08	94112.23	276059.1
B1Z1-60-3	27044.23	12256.45	176768.44	98455.93	120468.4
B2Z1-60-1	16493.54	20022.56	150144.97	55047.31	133134
B2Z1-60-2	22770.19	14927.68	216222.23	63038.93	192217.1
B2Z1-60-3	24784.20	16616.22	194457.10	83384.97	153769.8
B3Z1-60-1	—	—	—	—	—
B3Z1-60-2	22856.87	16469.25	148193.94	72852.39	116113.5
B3Z1-60-3	13988.54	12051.59	158178.95	58117.13	127483.4
KJ	—	—	98049.19	—	98049.19

It can be observed in table 5 that the design with oblique bracing increases the energy absorption of the integrated precast structure at both yielding and failure states. Therefore, the rebar bracing increases the energy absorption of the specimen at each line of defence. Furthermore, upon initiation of the second seismic line of defence, more energy absorption occurs than previously. According to the calculation results, the energy consumption upon initiation of the second line of defence significantly exceeds that of the lightweight recycled concrete-filled frame (KJ), which means that the force-bearing capacity of the steel frame and the steel skeleton of the wall has been fully used.

### 3.7. Force-bearing analysis of the inclined section

Successful results have been yielded from the studies on the calculation methods for the shear-bearing capacity of the inclined section of concrete shear wall both reinforced by structural steel and reinforced by steel plates. This study builds on the findings of the tests and refers to the calculation methods for the shear capacity of a concrete shear wall with a side frame, as stipulated in the *Code for Design of Composite Structures* (JGJ138-2016 [17]), the design code of an integrated structure. On this basis, it is concluded that three parts of the integrated precast structure contribute to the force-bearing capacity of the inclined sections. The three parts are the lightweight recycled concrete wall with single-row reinforcement, the lightweight steel frame columns filled with recycled concrete and the oblique bracing. The tests show that for the proposed design, shear cracks first appear on the wall. Before the applied loading reaches the peak value, the shear cracks have already started to extend largely. At this point, the wall and the steel frame work together in the task of load bearing, the force-bearing characteristic of the wall and steel frame can be categorized as that of a recycled concrete shear wall with single-row reinforcement restrained by recycled concrete-filled steel pipe frame. This cooperative mode of force bearing continues until the opening of the wall cracks, after which the performance of the wall deteriorates quickly and the frame bears the imposed loading alone. It is noted that because the wall and the frame jointly bear the force, the specimen exerts its shear resistance capacity to withstand the loading; therefore, the structure shows the sign of shear failure.

**Figure 14.**
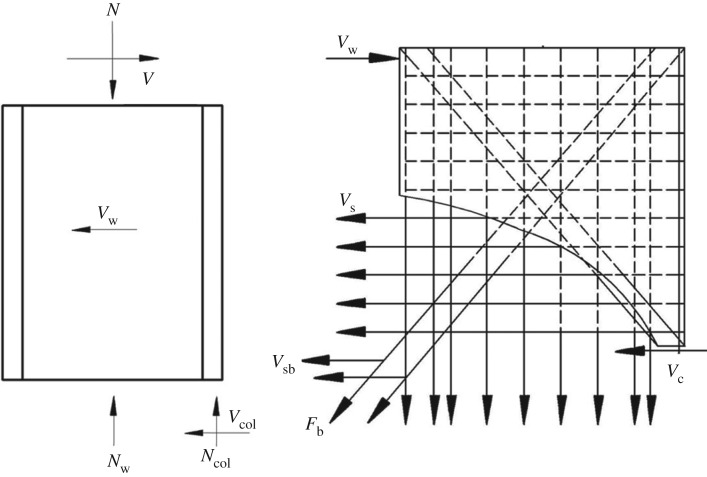
Calculation model for shear resistance.

Figure 14 and the following equation present the calculation model.

3.4 $$V=V_{w}+V_{\mathrm{col}},$$

where *V*_w_ is the horizontal shear resistance of the wall, and *V*_col_ is the horizontal shear resistance of the steel pipe frame column. *V*_w_ considers three aspects (figure 14): the shear force on the shear compression zone of the recycled concrete (*V*_c_), the shear resistance of the horizontally arranged rebar (*V*_s_) and the shear resistance of the oblique bracing (*V*_sb_). The equation below displays the relation among these parameters.

3.5 $$V_{w}=V_{c}+V_{s}+V_{\mathrm{sb}},$$

where

3.6 $$V_{c}=\frac{1}{\lambda -0.5}\left(0.05\beta_{r}f_{c}b_{w}h_{w0}+0.13N\frac{A_{w}}{A}\right),$$

3.7 $$V_{s}=f_{\mathrm{yh}}\frac{A_{\mathrm{sh}}}{s}h_{w0}$$

3.8 $$\;\text{and}\;V_{\mathrm{sb}}=f_{\mathrm{yb}}A_{\mathrm{sb}}\cos\theta ,$$

where the different parameters are as follows: *N* is the designed axial compression of the wall (when *N* > 0.2*f*_c_*b*_w_*h*_w_, *N* = 0.2*f*_c_*b*_w_*h*_w_); *λ* is the shear span ratio of the calculated section (when *λ* < 1.5, *λ* is rounded to 1.5, and when *λ* exceeds 2.2, it is set to 2.2); *β*_r_ is the restraint ratio of the steel pipe frame to the wall (*β*_r_ = 1.2 in this paper); *b*_w_ is the width of the wall section; *h*_w_ is the height of the wall section; *h*_w0_ is the effective height of the wall section; *A* is the area of the total cross-section of the wall; *A*_w_ is the web area of the cross-section of the wall; *f*_t_ is the axial tensile strength of the recycled concrete; *f*_yh_ is the tensile yield strength of the horizontally arranged rebars; *A*_sh_ is the section area of the horizontally arranged rebars at the same height; *S* is the vertical spacing of the horizontally arranged rebars; *f*_yb_ is the tensile strength of oblique bracing; *A*_sb_ is the area of the cross-section of the oblique bracing; *θ* is the inclination of the oblique bracing; *A*_a_ is the area of the steel pipe; and *f*_a_ is the tensile strength of the steel pipe.

The formula below is applied to calculate the shear capacity of the steel pipe frame columns filled with recycled concrete.

3.9 $$V_{\mathrm{col}}=\frac{0.4f_{a}A_{a}}{\lambda }.$$

The formula for the shear capacity of the oblique section of the integrated structure is obtained upon the substitution of equations (3.5)–(3.9) into equation (3.4).

3.10 $$V=\frac{1}{\lambda -0.5}\left(0.05\beta_{r}f_{c}b_{w}h_{w0}+0.13N\frac{A_{w}}{A}\right)+f_{\mathrm{yh}}\frac{A_{\mathrm{sh}}}{s}h_{w0}+f_{\mathrm{yb}}A_{\mathrm{sb}}\cos\theta +\frac{0.4f_{a}A_{a}}{\lambda }.$$

The shear capacity (*V*) values of all specimens are then calculated using equation (3.10). Table 6 presents the comparison of the calculated values and the measured values.

**Table 6.** Comparison of the calculated values and the measured values.

	calculated bearing capacity of the oblique section		
specimen no.	*V* (kN)	measured value *V*_u_ (kN)	relative error *η* (%)
B1Z1-60-1	1026.96	986.29	4.12
B1Z1-60-2	1037.87	1076.78	−3.61
B1Z1-60-3	1079.79	1166.75	−7.45
B2Z1-60-1	911.3	889.53	2.45
B2Z1-60-2	922.25	1023.84	−9.92
B2Z1-60-3	964.13	1045.74	−7.80
B3Z1-60-1	940.25	969.98	−3.07
B3Z1-60-2	951.16	973.77	−2.32
B3Z1-60-3	993.08	918.35	8.14

It can be concluded from table 6 that the calculation error of the oblique section ranges between 2.32 and 9.92%. This means that the formula proposed for the calculation of the bearing capacity of the oblique section has been proven to be highly relevant, and the results obtained are accurate.

## 4. Conclusion

(i) Amid the process of force bearing, the recycled concrete wall withstands the shear force, and the lightweight steel frame bears the vertical loading. The structure shows two distinct seismic lines of defence, with the first being the lightweight recycled concrete wall restrained by the lightweight steel frame and the second being the lightweight steel frame. In the case of seismic incidence, the first line plays an important role in sliding resistance, but its role gradually declines as the damage increases. Afterwards, the lightweight steel frame bears the loading. Because of the well-structured design, the integrated structure can absorb the seismic energy at each stage. Because of the rib-reinforced joints connecting the lightweight steel pipe beams and columns (filled with recycled concrete), the specimens exhibited better performance in terms of both bending resistance and distortion resistance at the beam–column joints.

(ii) The setting of the bracing leads to fuller curves. Because the bond strength between rebars and recycled concrete exceeds that between steel plates and recycled concrete, it can be concluded that the rebar bracing outshines its counterpart in energy consumption. In addition, the rebar bracing elevates the overall performance of the structure, which then translates into better ductility, and the plate-type bracing undermines the deformation performance of the structure. Therefore, the rebar bracing design stands out as a preferable choice.

(iii) The smaller spacing of reinforcement results in closer and more evenly distributed cracks, the width of which is evidently smaller. Therefore, the decrease in the reinforcement spacing leads to a surge in crack loading, yield loading and loading peak, and the widening of the spacing most affects crack loading. Exceedingly wide spacing can undermine both the integrity and the ductility of the overall specimens.

(iv) The average displacement angle between elastic–plastic layers of the integrated structure of precast lightweight steel frame–lightweight wall is 1/24, which means that the structure promises good performance in force bearing, ductility and energy consumption. In engineering application, 1/24 can be introduced as the critical controlling parameter for collapse resistance.

## Data accessibility

Our data are deposited at the Dryad Digital Repository: http://dx.doi.org/10.5061/dryad.cm41ff4 [18].
